# Patient-reported outcomes provide evidence for increased depressive symptoms and increased mental impairment in giant cell arteritis

**DOI:** 10.3389/fmed.2023.1146815

**Published:** 2023-05-12

**Authors:** Matthias Froehlich, Antonia Zahner, Marc Schmalzing, Michael Gernert, Patrick-Pascal Strunz, Sebastian Hueper, Jan Portegys, Eva Christina Schwaneck, Ottar Gadeholt, Andrea Kübler, Johannes Hewig, Philipp Ziebell

**Affiliations:** ^1^Department of Internal Medicine II, Rheumatology/Clinical Immunology, University Hospital Würzburg, Würzburg, Germany; ^2^Department of Psychology I, Institute of Psychology, University of Würzburg, Würzburg, Germany; ^3^MVZ Rheumatologie und Autoimmunmedizin, Hamburg, Germany; ^4^Rheumatologische Schwerpunktpraxis Würzburg, Würzburg, Germany

**Keywords:** giant cell arteritis, PRO, depression, mental impairment, SF-36, PHQ-9, VAS, polymyalgia rheumatica

## Abstract

**Objectives:**

The spectrum of giant cell arteritis (GCA) and polymyalgia rheumatica (PMR) represents highly inflammatory rheumatic diseases. Patients mostly report severe physical impairment. Possible consequences for mental health have been scarcely studied. The aim of this study was to investigate psychological well-being in the context of GCA and PMR.

**Methods:**

Cross-sectional study with *N* = 100 patients with GCA and/or PMR (GCA-PMR). Patient-reported outcomes (PROs) were measured using the Short Form 36 Version 2 (SF-36v2) and visual analog scale (VAS) assessment. Moreover, the Patient Health Questionnaire 9 (PHQ-9) was used in 35 of 100 patients to detect depression. To compare PROs with physician assessment, VAS was also rated from physician perspective. To assess a possible association with inflammation itself, serological parameters of inflammation (C-reactive protein [CRP], erythrocyte sedimentation rate [ESR]) were included.

**Results:**

In all scales of the SF-36v2 except General Health (GH) and in the physical and mental sum score (PCS, MCS), a significant impairment compared to the German reference collective was evident (MCS: d = 0.533, *p* < 0.001). In the PHQ-9 categorization, 14 of the 35 (40%) showed evidence of major depression disorder. VAS Patient correlated significantly with PHQ-9 and SF-36 in all categories, while VAS Physician showed only correlations to physical categories and not in the mental dimensions. Regarding inflammatory parameters, linear regression showed CRP to be a complementary significant positive predictor of mental health subscale score, independent of pain.

**Conclusion:**

PRO show a relevant impairment of mental health up to symptoms of major depression disorder. The degree of depressive symptoms is also distinctly associated with the serological inflammatory marker CRP.

## 1. Introduction

Giant cell arteritis (GCA) and polymyalgia rheumatica (PMR) are rheumatic diseases of people of older age (1). Both entities belong to a common spectrum of highly inflammatory diseases and are closely related (2, 3). While GCA is primarily a vasculitis of the large vessels, PMR is a mostly symmetrical inflammation of the extracapsular structures, primarily of the shoulders and pelvic girdle (4). Both conditions can occur isolated, overlapping or sequential (2, 5, 6). Patients frequently experience severe pain and also report unspecific symptoms such as disturbed night sleep, weakness, and malaise, lowering quality of life (7, 8). To date, structured data on psychological dimensions of well-being in the context of the GCA-PMR spectrum are scarce, especially how strongly the disease is associated with major depression disorder (9, 10). It is also unclear whether the inflammatory activity itself impacts the mental state and quality of life (summarized from here as “psychological impairment”) or whether the impact is due the unspecific variables such as pain and sleep disturbances. Isolated case reports exist on brain organic symptoms in the context of GCA (11–13) that may lead to psychological impairment. Therefore, the aim of the present cross-sectional study was to investigate health related quality of life (HRQoL), the amount of depressive symptoms, and major depression disorder in patients with initial diagnosis or recent relapse of GCA-PMR, using patient reported outcome measures (PRO). As we know from rheumatoid arthritis (RA) (14), disease activity is assessed differently by patients and physicians. The visual analog scale (VAS) is a widely used tool for assessment by patients and physicians in the context of GCA-PMR. Therefore, our aim was to investigate the VAS-based agreement between both, in particular, the value of the VAS for estimating HRQoL. To further investigate a possible association between inflammation and psychological impairment, we created a regression model using C-reactive protein (CRP) as an inflammatory protein.

## 2. Patients and methods

### 2.1. Patients

Hundred consecutive patients with initial diagnosis (*n* = 88) or recent relapse (*n* = 12) of a condition of the GCA-PMR spectrum were recruited by the Department of Rheumatology and Immunology of the University Hospital of Wuerzburg between May 2019 and January 2021. Informed consent was obtained from each participant. A total of *n* = 120 patients were studied, of whom *n* = 20 were excluded for further analysis because of incomplete data, i.e., questionnaires were not completely filled out or blood values were missing. The final sample size was *n* = 100. All patients met either the ACR 2022 classification criteria for GCA (15) or the 2012 provisional EULAR classification criteria for PMR (16) and were part of the “Wuerzburg GCA Registry.” A defined set of patient-related outcome measures was collected in all patients, namely VAS Patient, SF-36v2 and PHQ-9. Since the PHQ-9 was not originally collected from all participants, a sample size of only *n* = 35 could be collected specifically for the PHQ-9. Patients completed the questionnaires while they were in the hospital or outpatient clinic. They received a short briefing and then completed the questionnaires themselves without any time limit. Physicians evaluated VAS during the visit. Furthermore, serological inflammatory markers were collected (C reactive protein, erythrocyte sedimentation rate [ESR]), the VAS Physician and the Glucocorticoid Toxicity Index (GTI). The present study was designed as a cross-sectional study at new onset/recent relapse of GCA and/or PMR to investigate the impact of the disease on HRQoL and depression.

### 2.2. Methods

#### 2.2.1. Questionnaires

To assess HRQoL, the second version of the SF-36v2 was used. In addition, to examine depression symptoms, the depression-specific module of the Patient Health Questionnaire (PHQ), the PHQ-9, was added. In all cases, the German version of the questionnaires was used. To obtain an assessment of current GCA activity and disease-related limitations, both the patient and the treating physician were asked to provide an individual score on a visual analog scale: VAS Patient and VAS Physician.

##### 2.2.1.1. SF-36v2

The SF-36v2 (*QualityMetric Inc.*) assesses subjective well-being and health with 8 different scales and 2 summary scales for physical and mental health. The eight scales include general health (GH), physical functioning (PF), role physical (RP), role emotional (RE), social functioning (SF), bodily pain (BP), vitality (VT), and mental health (MH). In addition, summary scores for the mental (MCS) and for the physical components (PCS) can be calculated from these scales. Scores between 0 and 100 are possible for each scale, with a higher score representing greater well-being and better health. Both the SF-36v2 and its predecessor, the SF-36, are reliable and valid (Cronbach’s α = 0.81–0.94) (17, 18). For comparison with healthy controls, reference collectives exist that consider age, sex, and home country. For our study, the age-matched German reference collective was used.

##### 2.2.1.2. PHQ-9

The PHQ-9 is a well-established and economic questionnaire to assess depression and consists of 9 questions, each of which corresponds to a diagnostic criterion for major depression disorder according to DSM-IV (19). These include perceptions of pleasure in activities, depressed mood, sleep disturbances, feelings of low energy or fatigue, decreased or increased appetite, feeling like a failure, difficulty concentrating, slowed speech and slowed movements or a strong urge to move, and suicidal thoughts and self-harming behavior. The questionnaire assesses the past two weeks (20) and can be scored categorically to obtain a cut-off score that makes a diagnosis of depression likely or as a summative score to determine the severity of depressive symptoms (19). It can be useful for diagnostic purposes, but can also be used to analyze treatment progress (21). Responses are scored in ascending order of 0, 1, 2, or 3 points, resulting in a total score ranging from 0 to 27, with a higher score indicating a higher amount of symptoms (19). A major depression is likely if 5 or more items are answered with at least “more than half of the days” and one of these items is depressed mood or anhedonia (item 1 or 2). A classification as “other depression” can be made if 2–4 items are answered as described above. When the PHQ-9 is used to measure the severity of depressive symptoms, a classification is made into minimal (total score: < 5), mild (total score: 5–9), moderate (total score: 10–14), moderately severe (total score: 15–19), and severe symptoms (total score: ≥ 20). Good validity and reliability values have been demonstrated (Cronbach’s α = 0.89) (19, 22).

##### 2.2.1.3. VAS

In the context of GCA, the VAS represents a tool to numerically assess disease activity and disease burden, with 0 mm representing the minimum value and 100 mm representing the maximum value. This was rated from the perspective of the patient (VAS Patient) and in a separate VAS also from the perspective of the treating physician (VAS Physician) (23). The VAS was collected as a paper version. VAS scales are commonly used to measure subjective attitudes, pain, or moods because they are easy to implement and can reflect the continuous nature of the measured value (24). Good psychometric properties have been found in various situations (23, 25, 26).

#### 2.2.2. Statistical analysis

All statistical analyses were performed using IBM SPSS Statistics 27.0 (IBM Corp., 2020) and jamovi 2.2.5 (The jamovi project, 2022). Means and 95% confidence intervals per scale were calculated from the SF-36v2 and compared with a German age- and sex-matched norm sample. This norm study was conducted between 2008 and 2011 by the Robert Koch Institute and the results were published in 2013 (27). For the comparison, one-sample *t*-tests with a significance level of *p* < 0.05 and Cohen’s d as a measure of effect size were calculated for each scale, with d ≥ 0.2 indicating a small effect size, ≥ 0.5 indicating a medium effect size and ≥ 0.8, indicating a large effect size (28). The PHQ-9 was evaluated categorically and using the sum score according to the suggestions of the questionnaire authors described earlier. Again, the mean score was compared with a German age- and sex-matched norm study conducted between 2003 and 2008 (29), using a one-sample t-test with a significance level of *p* < 0.05 and calculating Cohen’s d as an effect size measure. Pearson correlations (Pearson’s r) were calculated to assess associations between SF-36v2, PHQ-9, VAS scores, and C-reactive protein, with *r* ≥ 0.1 indicating a weak correlation, ≥ 0.3 to ≤0.5 indicating a moderate correlation and > 0.5, indicating a large effect size (28). Finally, we investigated whether the detectable mental health symptoms are a consequence of bodily pain as a frequent trigger of mental health symptoms or whether they are independently associated with the inflammatory activity of the GCA. For this purpose, we performed a two-step hierarchical linear regression analysis using the bodily pain subscale of the SF-36v2 as a predictor and the SF-36v2 mental health subscale as the dependent variable and C-reactive protein (CRP) as an additional second-step predictor.

## 3. Results

### 3.1. SF-36v2

**Figure 1:**
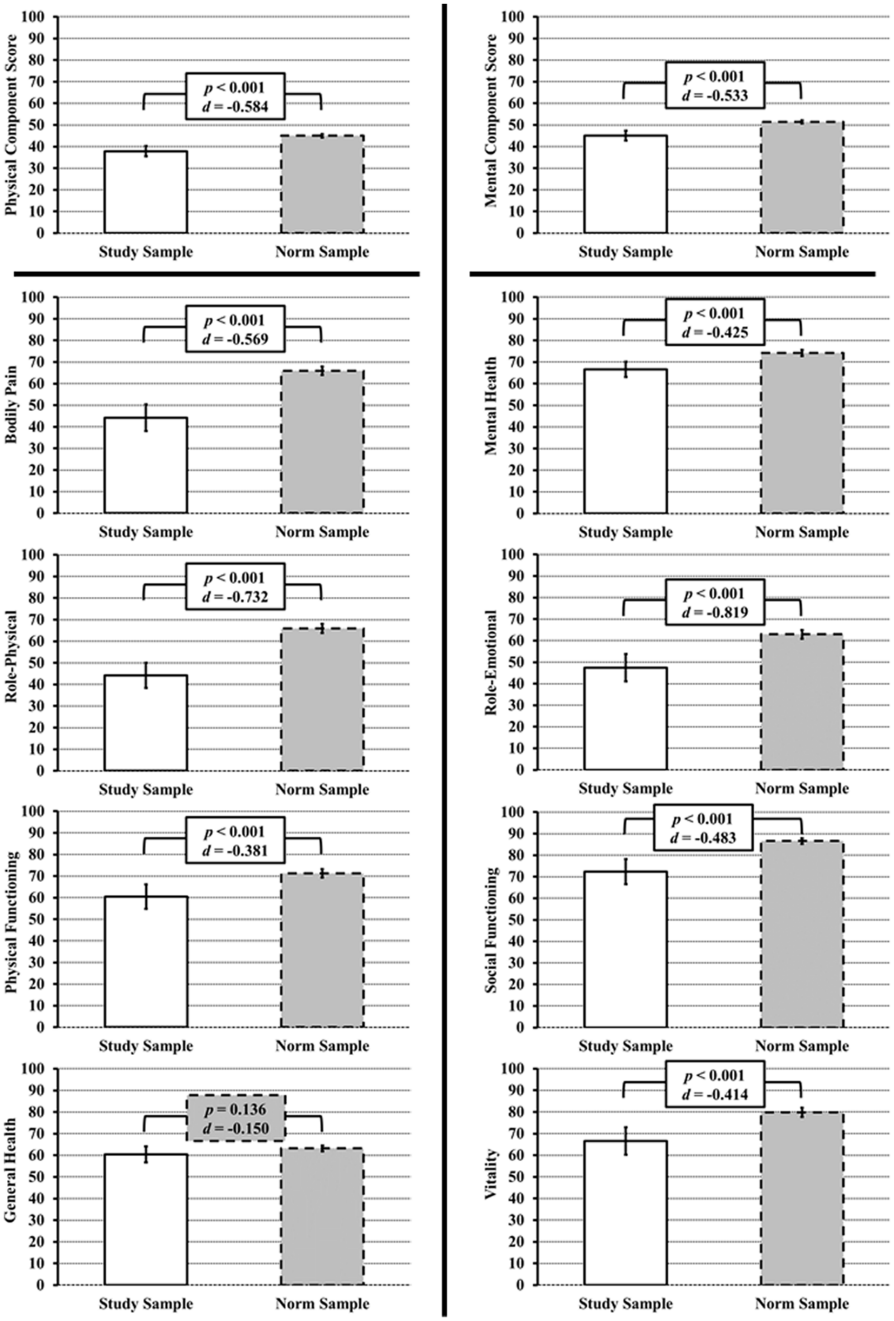
SF-36v2 scores at initial diagnosis/relapse of GCA and/or PMR. Left: Physical Component Score and all its subscales (Physical Pain, Physical Role, Physical Functioning) except General Health show a significantly lower score as compared to the SF-36v2 healthy norm sample (gray bars). Right: For the Mental Component Score and all its subscales (Mental Health, Role Emotional, Social Functioning, Vitality), a significantly lower score as compared to the SF-36v2 healthy norm sample is also evident.

The SF-36v2 questionnaire was collected from *n* = 100 patients of the “Würzburg GCA Registry.” The characteristics of the patients are summarized in Table 1. For the evaluation of the SF-36v2 questionnaire, the mean values were compared to the mean values of the German norm sample. With the exception of the GH dimension (*p* = 0.14), all other 7 scales of the SF-36v2 showed a significant decrease in score (*p* < 0.001 for all 7 scales) compared to the control sample, corresponding to a decrease in well-being in both the physical and mental dimensions. In the categories RE, RP the effect size was strong, in the remaining categories the effect size was moderate. The component scores, PCS and MCS, were also significantly reduced with moderate effect size (*p* < 0.001 for both, PCS d = −0.58, MCS d = −0.53). The results are shown in Figure 1 and Supplementary Table S1.

**Table 1:** Patients’ characteristics at time of evaluation.

Variable	Total *N* = 100
Clinical parameters	
Female	61 (61%)
Age (years)	71.5 ± 8.5
Giant cell arteritis	66 (66%)
Polymyalgia rheumatica	25 (25%)
Giant cell arteritis/polymyalgia rheumatica	9 (9%)
New diagnosis	88 (88%)
Relapse	12 (12%)
Therapy-naïve patients at time of assessment	28 (28%)
Dose of steroids at time of assessment	40 ± 149 mg
Patients on immunosuppressants	14 (14%)
Markers of inflammation	
C reactive protein (CRP)	2.2 ± 2.94 mg/dl
Erythrocyte sedimentation rate (ESR)	30.67 ± 26.53 mm/1st hour (*N* = 92)

Percentages are given in parentheses. Standard deviations reported for age, dose of steroids at time of assessment, C reactive protein, and erythrocyte sedimentation rate.

### 3.2. PHQ-9

**Figure 2:**
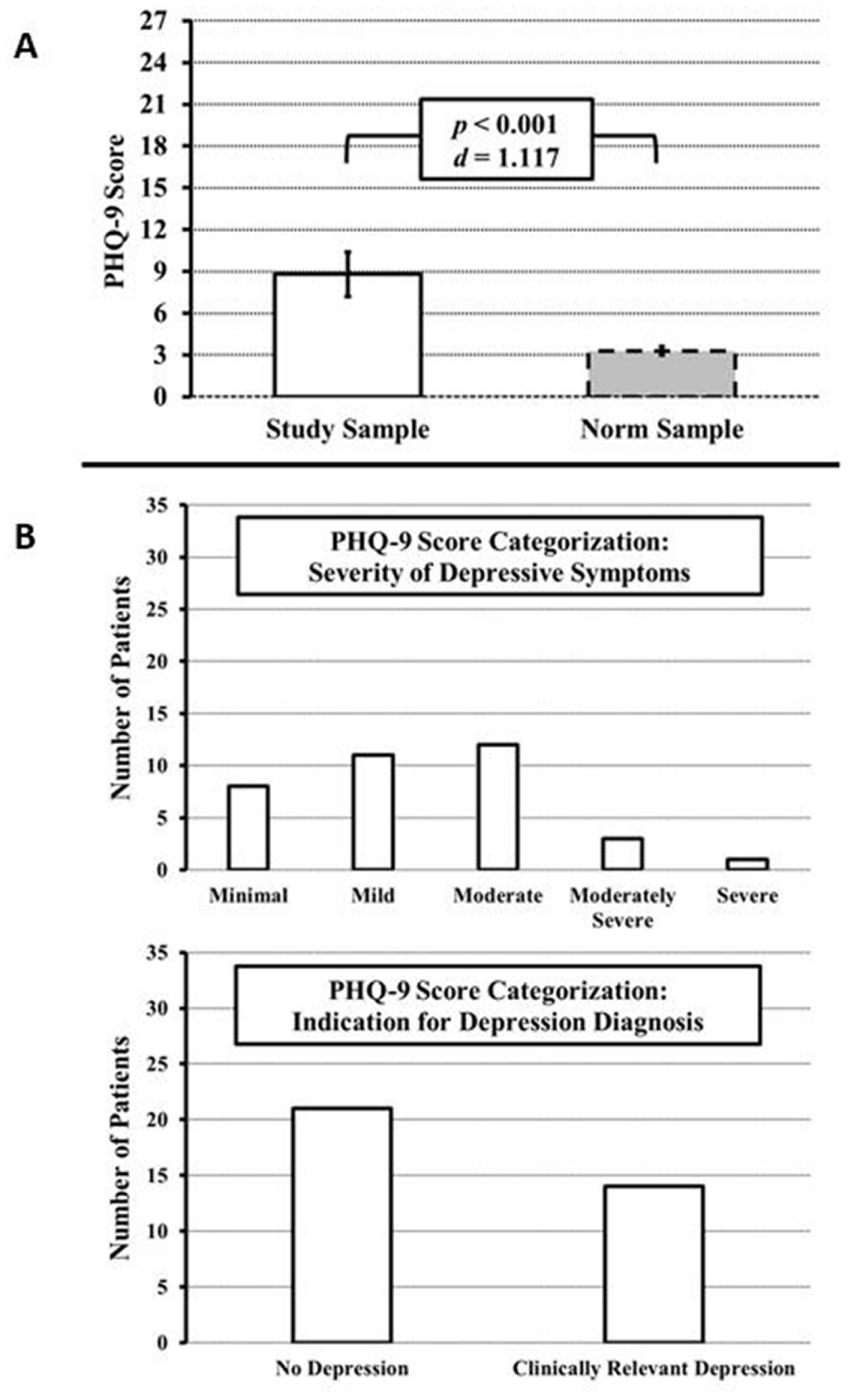
PHQ-9 scores at initial diagnosis or at relapse of GCA and/or PMR to detect depressive symptoms. **(A)** Compared to the German PHQ-9 healthy norm sample, the prevalence of depressive symptoms is significantly increased. **(B)** PHQ-9 categorization approach. Top: Distribution by severity of depressive symptoms (minimal to severe). Bottom: Classification into non-clinically relevant and relevant depression according to Kroenke et al. (19).

The PHQ-9 questionnaire was collected in 35 of 100 patients in addition to the SF-36v2. Evaluation of the PHQ-9 yielded a mean score of 8.8 (SE = 0.82), which was significantly above normal (M = 3.3, SE = 0.13), *t*(35) = 6.606, *p* < 0.001, d = 1.117. In addition, the PHQ-9 was evaluated according to the severity of symptoms (Figure 2). Minimal symptoms were present in 8 subjects (22.86%), mild symptoms in 11 subjects (31.43%), moderate symptoms in 12 subjects (34.43%), moderately severe symptoms in 3 subjects (8.57%), and severe symptoms in 1 subject (2.86%). Overall, 77.14% of patients had values above the cut-off value of ≥5 (at least mild symptoms) and 45.71% of patients had symptoms above the cut-off value of ≥10 (at least moderate symptoms).

According to the PHQ-9 categorization approach, 8 subjects met the proposed criteria for major depression (22.86%) and 6 subjects met the criteria for other depression (17.14%) (Figure 2). Thus, in total, 14 (40%) of the 35 patients had depressive symptoms that were categorized as clinically relevant or in other words, relevant to consider for depression-specific clinical interventions.

### 3.3. VAS

The mean value of the VAS Patient was 42.8 ± 28.4 mm, the mean value of the VAS Physician was 32.8 ± 31.5 mm. Thus, the assessment of disease activity differed significantly between patients and physicians (*p* = 0.003). In no VAS Patient and no VAS Physician was activity reported as 0 mm, i.e., according to patient and physician assessment, disease activity was present in all patients at the time of assessment.

### 3.4. Correlations of SF-36v2, PHQ-9, and VAS

Some of the items of the mental components of the SF-36v2 refer to depressive symptoms, but unlike the PHQ-9 it does not explicitly examine depression. Therefore, a comparison of SF-36v2, PHQ-9 and VAS Patient and VAS Physician was made. The results are shown in Table 2. All individual scales of the SF-36v2 showed significant negative correlations with the sum of the PHQ-9; accordingly, the higher the depression in the PHQ-9, the lower the well-being reported in the SF-36v2. The sum scores (PCS and MCS) also showed a strong negative correlation, although this was more pronounced for the MCS than for the PCS: MCS: *p* < 0.001, *r* = −0.79, PCS: *p* < 0. 001, *r* = −0.57.

**Table 2:** Correlations of SF-36v2, PHQ-9, and VAS.

		PHQ-9	VAS Physician	VAS Patient	PCS	MCS
PHQ-9	Pearson’s r	—				
	value of *p*	—				
	*N*	—				
VAS Physician	Pearson’s r	0.131	—			
	value of *p*	0.491	—			
	*N*	30	—			
VAS Patient	Pearson’s r	0.414	0.472	—		
	value of *p*	0.017	<0.001	—		
	*N*	33	97	—		
PCS	Pearson’s r	−0.572	−0.455	−0.618	—	
	value of *p*	<0.001	<0.001	<0.001	—	
	*N*	35	93	97	—	
MCS	Pearson’s r	−0.795	−0.032	−0.255	0.296	—
	value of *p*	<0.001	0.762	0.012	0.003	—
	*N*	35	93	97	100	—

The SF-36v2 sum scores (PCS and MCS) show a significant negative correlation with the PHQ-9 with moderate to strong effect sizes ranging from *r* = −0.408 to *r* = −0.802. The VAS Patient correlates significantly with the VAS Physician.

The VAS Patient showed a significant negative correlation of medium size with the PHQ-9 score and significant negative correlations of small to strong effect sizes with the SF-36v2, both in the individual categories and the sum scores. In contrast, the VAS Physician showed a significant negative correlation of medium size only in the physical categories of the SF-36v2. No correlation was detectable in the mental categories, respectively MCS, of the SF-36v2 as well as the PHQ-9. VAS Patient and VAS Physician showed also a significant positive correlation with moderate effect.

### 3.5. Prediction of mental health symptoms based on bodily pain and CRP

In the first step, the bodily pain subscale score could be shown to be a significant positive predictor for the mental health subscale score. Adding CRP as an additional predictor in the second step of the hierarchical regression could significantly explain additional unique variance, with CRP as a complimenting significant positive predictor for the mental health subscale score. The results are summarized in Table 3.

**Table 3:** Overview of the hierarchical linear regression analysis for the prediction the SF-36v2 subscale mental health, based on the SF-36v2 subscale bodily pain (including 100 patients) and CRP (including 98 patients).

Model 1: *R^2^ =* 0.178	
Single predictor	0.237 [0.052]
*B* (bodily pain) [*SE*]	0.422
*Beta* (bodily pain)	*p* < 0.001
Model 2: *R^2^ =* 0.237	
1st step predictor	0.306 [0.056]
*B* (bodily pain) [*SE*]	0.545
*Beta* (bodily pain)	*p* < 0.001
2nd step predictor	1.650 [0.607]
*B* (CRP) [*SE*]	0.273
*Beta* (CRP)	*p* = 0.008
Model 1 vs. Model 2: ΔR^2^ = 0.059	
*p* = 0.008	

*B* = unstandardized regression coefficient. SE = standard error. Beta = standardized regression coefficient. *R^2^* = explained variance.

## 4. Discussion

The aim of the study was to shed light on the relationship between GCA-PMR and impairment of mental health, here defined as the degree of depression symptoms and health related quality of life. Our results support the assumption that patients with GCA-PMR have worse HRQoL, higher depression scores and are in general not only physically but also mentally affected. These results are in line with recent studies, whereas the proportion of patients with depressive symptoms is significantly higher in our sample, possibly due to the additional application of the specific depression questionnaire PHQ 9 in a sub-cohort (30). Examination of the 8 scales of the SF-36v2 and the 2 component scores revealed that our collective was significantly below normal in all scales except the GH scale, with moderate to strong effect sizes (Cohen’s d). This shows that the disease can affect all areas of HRQoL.

Of note, regression analysis showed also that mental impairment correlates with the inflammatory disease activity as measured by the CRP level. This suggests that inflammatory activity itself has an impact on psychological well-being, especially since several studies have associated higher blood CRP levels with more severe depression symptoms and poorer response to antidepressant treatment (31). This association has also recently been demonstrated in patients with rheumatoid arthritis (RA) and concurrent depression (32, 33).

Our results further demonstrate depressive symptoms as prevalent. PhQ-9 scores of patients in our sample were clearly above the norm (≥ 10) and about 46% presented with moderate to severe symptoms of depression. In the comparative group of the German norm study, this value was found only in about 6% of the participants in all age groups (29). This score is often used as cut-off to decide whether further examinations such as a clinical-psychological interview should be conducted to validate the diagnosis of major depression (34). In a study of the authors of the PHQ-9, a cut-off score of ≥10 showed a sensitivity and specificity of 88% for major depression (19). Using the categorical evaluation of the PHQ-9, 40% of the participants in our study met the proposed criteria for major- or other depression.

In line with the previous studies mentioned above, correlations between the PHQ-9 and all scales of the SF-36v2 were also found in the present study, including the MH, VT, and MCS scales, which are relevant for the assessment of depressive symptoms. Consequently, both questionnaires are useful in assessing depressive symptoms.

Physicians’ assessment of disease activity using the VAS correlated well with the physical dimensions of the SF-36v2 and with patients’ VAS. However, similar to other rheumatologic entities, disease activity was rated significantly lower by the physician than by the patient (14, 35). With respect to the mental categories of the SF-36v2 and the PHQ-9, a significant discrepancy was demonstrated between the VAS physician and the PROs. No correlations were found. This suggests that mental impairment is underestimated by the physicians, especially since the VAS patients showed significant correlations, suggesting that the VAS is an appropriate instrument for assessing mental dimensions. Nevertheless, the direct comparison of VAS Patient and VAS Physician should be evaluated with caution, because the assessment is purely subjective and the respective assessment basis of the VAS is not precisely defined. Patient-reported outcome measures (PROs) give patients the opportunity to evaluate their current health status and the effect of a therapeutic intervention, taking their individual needs into account (36). The perspective of the treating physicians and nurses is thus supplemented by the patient’s view and PROs can therefore offer a deeper insight into the success of the therapy and the patient’s current quality of life (37). Furthermore, active involvement of the patients and continuous monitoring can generally improve adherence and communication between patient and physician and has also been associated with fewer reported symptoms of depression during treatment (38–40). Overall, PROs in combination with clinical data (blood count, imaging techniques, etc.) can provide a comprehensive picture of current well-being and the impact of interventions. There is no specific PRO for GCA-PMR yet, but it is currently developed by the Outcome Measurement in Rheumatology (OMERACT) Vasculitis Working Group (41). Patients with GCA were asked about important aspects of their HRQoL and possible items were developed from these: The result is a draft questionnaire, with psychometric testing still pending, that includes questions on topics such as: Fear of potential blindness, fatigue, pain, difficulty in socializing and despondency (42). A GCA-PMR-specific PRO could refine the course of treatment by better understanding patients’ concerns. Perhaps we could find out whether there are different subgroups in the spectrum of GCA and PMR in relation to the recognition that both diseases belong to a spectrum of inflammatory diseases with different clinical manifestations (2). Robson and colleagues recently published a first draft for a GCA specific PRO-set, that now has to be further evaluated (42). In the case of GCA-PMR, PROs could be used to screen for depressive symptoms and then, if necessary, provide additional psychological treatment or referral to a psychiatric department.

A limitation of this study is that depressive symptoms were only assessed in a sub-cohort by the PHQ-9 questionnaire. Therefore, to solidify the interpretation of the PHQ-9, scatterplots of its correlations with the VAS and SF-36v2 scales are attached as a Supplementary Figure S1. Supplementary Figure S1 also includes scatterplots for the regression analysis variables as well as for the PCS with its subscales and the MCS with its subscales. Visual analysis of these scatterplots supports the idea that the significant correlations were not caused by outliers. However, the subpopulation that additionally used the PHQ-9 questionnaire did not have any special characteristics that could influence the results. Another limitation is that glucocorticoids may have effects on psychological well-being (43). 28 patients were treatment-naive at the time of the study; all other patients had glucocorticoid treatment. However, we demonstrated that psychological impairment was related to inflammation, although the treatment itself might have an impact on psychological well-being, this should be calculated with, as glucocorticoids are the cornerstone of GCA-PMR therapy. Future work is planned to extend the reported results with a larger sample and to evaluate the course of treatment, as well as to define different subgroups of patients, first, to identify those patients who need special care such as psychological intervention and, second, to possibly associate specific symptoms or conditions with psychological impairment, e.g., via a network analysis approach [such as for example Schuler et al. (44)], provided for COPD and depression (44) that however, would need a considerably large sample size to reveal valid information [in the example of Schuler et al., (44), *N* = 590].

## 5. Conclusion

Symptoms of depression up to clinically relevant depression disorders are highly probable in patients with GCA-PMR and are probably linked to inflammation itself. The SF-36v2 is a good instrument to detect physical and mental impairment and correlates highly with the PHQ-9 as a depression-specific questionnaire. On the other hand VAS physician is not suitable to adequately assess depressive symptoms in patients with GCA-PMR. The underestimation of mental symptoms by physicians strengthens the current view that GCA-PMR specific PROs are mandatory to assess comprehensively patients’ well-being.

## Data availability statement

The original contributions presented in the study are included in the article/Supplementary material, further inquiries can be directed to the corresponding author.

## Ethics statement

The studies involving human participants were reviewed and approved by Ethics Committee of University of Wuerzburg (Code: 109/19-am, Date: 25.04.2019). The patients/participants provided their written informed consent to participate in this study.

## Author contributions

MF and PZ: conceptualization and project administration. MF, AK, and PZ: methodology. MA and AZ: software and formal analysis. MS, MG, and P-PS: validation. MF, AZ, P-PS, SH, JP, and PZ: investigation. MZ and AK: resources. MF, AZ, and PZ: writing—original draft preparation. MS, MG, SE, AK, JH, and MS: writing—review and editing. MF, AZ, and PZ: visualization. MS, AK, and JH: supervision. All authors have read and agreed to the published version of the manuscript.

## Funding

This publication was supported by the Open Access Publication Fund of the University of Wuerzburg.

## Conflict of interest

MF received speaker’s fees, travel grants or compensation for board memberships from AbbVie, Novartis, Janssen, and Eli Lilly. MS received speaker’s fees, travel grants, research funding, or compensation for consultancies or board memberships from AbbVie, Actelion, AstraZeneca, BMS, Boehringer/Ingelheim, Celgene, Chugai/Roche, Eli Lilly, Genzyme, Gilead, Hexal/Sandoz, Janssen-Cilag, MSD, Novartis, Pfizer, Sanofi Pasteur, Takeda (Shire), UCB (less than $ 10,000 each). MG received travel grants, compensation for advisory boards or speaker’s fees from AbbVie, Chugai, Eli Lilly, Hexal, Janssen, Novartis, Pfizer, Takeda. P-PS received travel grants from AbbVie and Janssen-Cilag. JP received travel grants from CSL Behring, Galapagos, AbbVie. SE received speaker’s fees, travel grants, research funding, or compensation for consultancies or board memberships from Janssen, Eli Lilly, Chugai, Roche, Takeda (Shire), AbbVie, Novartis, Pfizer, Galapagos, Amgen.

The remaining authors declare that the research was conducted in the absence of any commercial or financial relationships that could be construed as a potential conflict of interest.

## Publisher’s note

All claims expressed in this article are solely those of the authors and do not necessarily represent those of their affiliated organizations, or those of the publisher, the editors and the reviewers. Any product that may be evaluated in this article, or claim that may be made by its manufacturer, is not guaranteed or endorsed by the publisher.

## Supplementary material

The Supplementary material for this article can be found online at: https://www.frontiersin.org/articles/10.3389/fmed.2023.1146815/full#supplementary-material
