# Supplementary material for: Patient-reported outcomes provide evidence for increased depressive symptoms and increased mental impairment in giant cell arteritis
Source: Front Med (Lausanne). 2023 May 12;10:1146815. doi: 10.3389/fmed.2023.1146815 (PMC10262919; doi:10.3389/fmed.2023.1146815)
Supplement: Supplementary file 1 [file Data_Sheet_1.docx]

**Supplement**


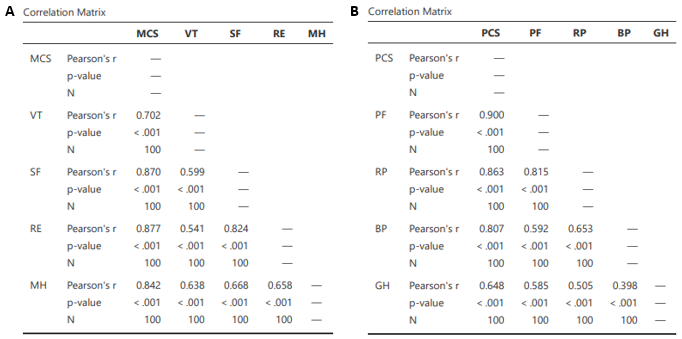


**Table S1:** Correlations of the SF-36v2 sum scores and the individual categories: (A) Mental categories and corresponding sum score, (B) physical categories and corresponding sum score. All individual categories show a significant positive correlation with the associated sum scores with strong effect size. This indicates that the sum score give a representative overview of patients‘ physical and mental well-being or impairment, respectively. MCS= Mental component score, VT = vitality, SF = social functioning, RE = role emotional, MH = mental health, PCS = physical component score, PF = physical functioning, RP = role physical, BP = bodily pain, GH = general health.


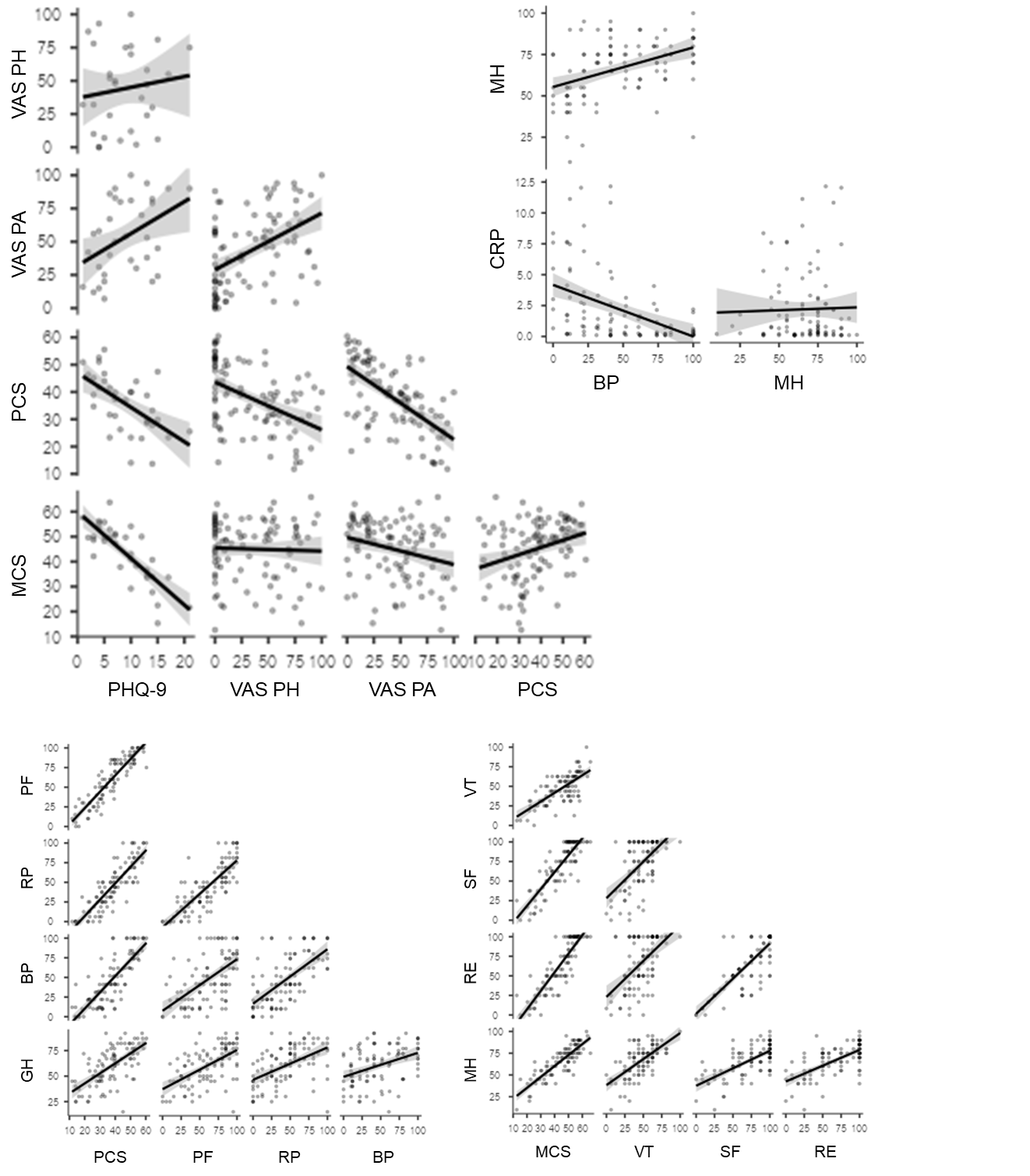


**Figure S1** Visual analysis supports the idea that the significant correlations were not caused by outliers. Upper left: Scatterplots for the correlations of the PHQ-9 score with VAS and SF-36v2 for all 35 participants who filled out the PHQ-9. Upper right: Scatterplots for regression analysis variables. Lower left: Scatterplots for PCS and its subscales. Lower right: Scatterplots for MCS and its subscales.
